# Persistently elevated alpha-fetoprotein associated with chronic hepatitis B during chemotherapy for malignant ovarian germ cell tumors: a case series and a review of the literature

**DOI:** 10.1186/s13048-019-0598-x

**Published:** 2019-12-13

**Authors:** Xuan Zong, Jia-Xin Yang, Ying Zhang

**Affiliations:** Department of Obstetrics and Gynecology, Peking Union Medical College Hospital, Chinese Academy of Medical Sciences and Peking Union Medical College, No. 1 Shuaifuyuan, Dongcheng District, Beijing, 100730 China

**Keywords:** Germ cell tumor, Alpha-fetoprotein, Chronic hepatitis B, Liver dysfunction, Ovarian cancer

## Abstract

**Background:**

Alpha-fetoprotein (AFP) plays a crucial role in the management of malignant ovarian germ cell tumors (MOGCTs) and is an important reference index for chemotherapy termination. However, a high level of AFP can also be caused by several benign diseases, causing confusion and impacting treatment decisions.

**Case presentation:**

We described four patients who were diagnosed with MOGCTs; the histologic subtype in two of them was mixed MOGCTs (yolk sac tumor with mature teratoma), while the rest was immature teratoma. The serum AFP level of each patient was abnormal before surgery, but it was still persistently elevated around 300 ng/ml even after additional cycles of chemotherapy. All patients were thoroughly evaluated, but we did not find any evidence of disease progression or residual tumors. Liver function tests were normal, whereas serum assays revealed positive of hepatitis B surface antigen, and two patients had a high level of HBV-DNA. They were chronic carriers of hepatitis B virus and never received relevant treatments. Then they were managed with tumor surveillance and the antiviral treatment. Thereafter, the AFP levels presented a slowly decreasing trend.

**Conclusions:**

False elevation of AFP in MOGCTs is a rare condition and should be assessed with a comprehensive evaluation to avoid unnecessary treatments.

## Background

Malignant ovarian germ cell tumors (MOGCTs) mostly occur in adolescent and young adults under the age of 35, and encompasses several histological subtypes, including dysgerminoma (DG), yolk sac tumor (YST), and immature teratoma (IT) [1, 2]. Treatment includes surgery and timely chemotherapy [3]. Since the 1980s, the prognosis of MOGCTs has greatly improved due to the use of cisplatin-based multi-agent chemotherapy, and the 5-year survival rate is more than 85% after standard management [4]. Specifically, the combination therapy of cisplatin, etoposide, and bleomycin (PEB) has become the first-line chemotherapy regimen and most patients with MOGCTs, except for those with stage I DG or stage I/grade 1 IT, are recommended to receive postoperative chemotherapy [5, 6]. As for the surgical approach, regardless of the disease stage of the patient, fertility-sparing surgery is mostly performed [7]. However, the need for comprehensive surgical staging, especially systematic lymphadenectomy, is still debated [8–10]. The frequency of lymph node metastasis in MOGCTs at first diagnosis is low and the results of recent studies have not demonstrated an association between lymphadenectomy in primary surgery and survival benefit [11–14]. Therefore, as our previous study recommended, instead of lymphadenectomy and omentectomy, the excision of only grossly abnormal lymph nodes and omental lesions is performed at our medical center [9].

Due to the relative rarity of the tumor, there are still several treatment challenges and pitfalls in the management of germ cell tumors (GCTs) [15], One common pitfall is that standard first-line chemotherapy regimen is not applied or doses of the drugs are not adequate [16]. Secondly, patients failed to be referred to and treated in experienced medical centers [17]. What’s more, a frequent mistake made during management is inappropriate use of examinations, including imaging technologies and tumor markers. Before altering treatment decisions, non-GCTs-related causes of tumor marker elevation should be considered [18, 19].

Alpha-fetoprotein (AFP) is one of the translational products of the family of albuminoid genes and is characterized as an embryo-specific and tumor-associated glycoprotein [20, 21]. During fetal development, AFP is mainly produced by the yolk sac and the fetal liver [22]. In normal adults, serum AFP level is found at very low concentrations [23]. The AFP level increases in certain malignancies, especially in GCTs and hepatocellular carcinoma (HCC) [24, 25]. Elevated AFP, usually less than 100 ng/ml, can also be observed in benign diseases, with abnormal liver function being the most common associated condition [26, 27]. The AFP derived from different tissues and diseases differs in carbohydrate moieties, which can be detected by the binding capacities of the lectins [28, 29]. AFP-L3 is one of the subtypes and most commonly detected in malignant conditions [30].

In MOGCTs, serum AFP level is predominantly elevated in YST and decreases rapidly during the chemotherapy [1]. Failure in decline of AFP is usually considered a sign of chemoresistance or existing residual tumors, which requires further treatments and predicts poor prognosis [4, 13]. However, the specificity of AFP is limited, and false positives do occur. In such circumstances, it can be difficult to correctly interpret the persistent elevation of AFP. As a result, some patients might be overtreated. To better understand this rare situation, we report four cases with false elevations of AFP in patients with MOGCTs.

### Case presentation

#### Case 1

A 29-year-old woman presented with a pelvic mass and underwent an open laparotomy exploration at a local hospital. The intraoperative visualization showed a 15-cm left adnexal mass without ascitic fluid and other suspicious metastatic nodes. The encapsulated mass was completely removed, and frozen-section pathological analysis revealed a mature teratoma with some glands that displayed atypical hyperplasia. Considering that the patient had a strong desire to preserve fertility, only left salpingo-oophorectomy was performed. The postoperative pathological analysis revealed mixed germ cell tumors (YST with mature teratoma). The patient received 3 cycles of PEB chemotherapy (cisplatin 40 mg daily on days 1–3; etoposide 150 mg daily on days 1–3; bleomycin 15 mg daily on days 1–2). The serum AFP level (normal range: 0–20 ng/ml) was ≥1200 ng/ml before chemotherapy and it was 350.2 ng/ml, 240.4 ng/ml, and 245 ng/ml after each cycle of chemotherapy, respectively.

Owing to the unsatisfactory decline in the serum AFP level, the patient was referred to our hospital. A pathology consultation confirmed the diagnosis. The serum AFP level had increased to 313.7 ng/ml, and positron emission tomography/computed tomography (PET/CT) showed one increased radioactivity uptake focus with an SUVmax of 2.5 located in front of the left psoas muscles at the level of the sacroiliac joint. She underwent laparoscopic exploration and left pelvic lymph node dissection. Intraoperative observation revealed severe adhesion that attached the sigmoid colon and rectum to the posterior uterine wall. A second-look surgery did not disclose any residual tumors. After these procedures, the patient received 2 cycles of PEB chemotherapy (cisplatin 50 mg daily on days 1–3; etoposide 150 mg daily on days 1–3; bleomycin 22.5 mg daily on days 1–2), and the AFP measurements taken on the 20th day after each cycle were 273.4 ng/ml and 289.9 ng/ml, respectively.

Considering the persistent elevated AFP levels, we performed a comprehensive evaluation. The liver function test was normal, but serum assays were positive for both hepatitis B surface antigen (HBsAg) and hepatitis Be antigen (HBeAg) and showed a high level of HBV-DNA. She was diagnosed with active viral hepatitis, and antiviral therapy was initiated following the physician’s recommendation. In addition, a close follow-up protocol instead of further chemotherapy was agreed upon with the patient. The AFP level declined to 146.3 ng/ml 2 months after the final treatment and continued to decrease. At the last follow-up, the patient was alive and without disease for 11 months, and the AFP level was 17 ng/ml.

#### Case 2

A 34-year-old woman underwent emergency exploratory laparotomy at a local hospital. An 11-cm right adnexal mass was observed intraoperatively, and right adnexectomy was performed. Postoperative pathological analysis revealed an immature teratoma (grade 2). Since the patient had no desire to maintain fertility, a staging surgery was performed, and no other tumors were found. After the second surgery, the patient underwent 5 cycles of PEB chemotherapy. The AFP level before the first surgery was 14.71 ng/ml, and it gradually increased after each cycle, reaching to 188.2 ng/ml after the fifth cycle.

The patient was referred to our hospital, and the AFP level had increased to 294.5 ng/ml. A complete examination was carried out with the following results: PET/CT did not show any suspicious lesions; serum tests revealed chronic hepatitis B with a normal level of HBV-DNA; and liver function tests were normal. The physician recommended that the patient initiate antiviral therapy and not continue any treatment associated with GCTs. The AFP level increased to 370.7 ng/ml 2 months after the final round of chemotherapy and then started to drop slowly. At the last follow-up, the patient was alive and without disease for 8 months, and the AFP level was 151 ng/ml.

#### Case 3

A 38-year-old woman underwent right adnexectomy at a local hospital, and the postoperative pathological analysis revealed an immature teratoma (grade 1). The patient underwent 1 cycle of PEB chemotherapy due to the high levels of preoperative AFP (289.8 ng/ml). However, AFP was still high at 185.9 ng/ml after the treatment. Then she was referred to our hospital.

A thorough examination did not find any evidence of residual tumors. The patient was diagnosed as a chronic carrier of HBsAg for 5 years without receiving any relevant treatment. The serum assays in our hospital revealed normal HBV-DNA and liver functions, and HBeAg was not detectable. We did not recommend any further tumor-related treatment. She has been in remission for more than 2 years and the AFP level has already been within the normal range.

#### Case 4

A 20-year-old woman underwent laparotomy at a local hospital, and intraoperative analysis revealed tumors on the left ovary and omentum majus. Left adnexectomy and omentectomy were performed, and the pathological analysis revealed that both tumors were mixed germ cell tumors (YST with mature teratoma). FIGO stage was III. The AFP decreased from preoperative 46,517 ng/ml to postoperative 346 ng/ml. However, the patient refused chemotherapy.

After 4 months, AFP had increased to 55,029 ng/ml, and PET/CT showed multiple metastatic neoplasms. She received 3 cycles of PEB chemotherapy at a local hospital, and was then referred to our hospital. AFP was still abnormal at 237 ng/ml. After a fully evaluation, we performed cytoreductive surgery, and pathological analysis confirmed that most tumors were necrosed, while YST could be found in a small part. After that, she received 6 cycles of chemotherapy including 4 cycles for consolidation. However, the AFP level was elevated again after ceasing chemotherapy for 2 months. PET/CT showed one increased radioactivity uptake focus around the spleen. Then, we performed the second cytoreductive surgery (splenectomy), and switched to a second-line combined therapy regimen of paclitaxel, ifosfamide and cisplatin (TIP). The patient received a total of 6 cycles, and the AFP level was maintained within the normal range after the second cycle. At the beginning of the follow-up period, her liver function tests appeared abnormal: alanine transaminase (ALT), 231 U/L (normal range: 7–40 U/L); Aspartate Transaminase (AST), 1079 U/L (normal range: 13–35 U/L). However, other blood indexes were normal, except for HBsAg positivity. She received oral liver protective drugs, and the AFP level was closely monitored. One month later, AFP was mildly elevated to 25.7 ng/ml, and increased to 117 ng/ml a month after that. Further examination revealed a high level of HBV-DNA, while ALT and AST had decreased to the normal ranges. The percentage of AFP-L3 tested at another hospital was < 5%. Taking all the results of assisted examinations into consideration, we did not consider that the increased AFP level was caused by tumor recurrence. Thus, we recommend the patient initiate the antiviral treatment and continue with ongoing surveillance. One months later, AFP decreased to 87 ng/ml, and the patient was still in the close follow-up period.

### Review of the literature

We searched the PubMed database for previous literature reports of false elevations of AFP in GCTs published in English between January 1, 1970 and December 31, 2018, using the search terms (“Neoplasms, Germ Cell and Embryonal” [MeSH] AND “AFP” [All Fields] AND “elevation” [All Fields]). Overall, 92 citations were identified. We screened all abstracts and included three articles [26, 31, 32]. Then, we reviewed the references included in these three articles to identify additional studies, and another six articles were included [18, 33–37]. Finally, we included a total of nine studies with samples sizes ranging from one to ten patients. All nine studies reported patients with seminoma, and four of the studies also reported patients with non-seminomatous GCTs. The detailed information is summarized in Table 1 and Table 2.

**Table 1:** Literature review of all previous cases of false elevations of AFP in non-seminomatous GCTs

Author/Year of publication	Case	Age	Histologic type	Stage	Maximum AFP level (ng/ml)	Etiology	Additional treatment	Outcome
Germa et al. 1993 [26]^a^	1^e^	26	YST	III	122	HD (anesthetic drugs)	Second surgery	ANED
	2	28	S, EC, YST	III	16	HD (HCV, alcohol)	–	ANED
	3	29	EC, T	II	91	HD (chemotherapeutic drugs)	–	ANED
	4	17	EC, T, YST	IV	42	HD (chemotherapeutic drugs)	–	ANED
	5	30	EC	II	18	HD (antiepileptic drugs)	–	ANED
	6	27	S, EC, YST	II	155	HD (HBV)	–	ANED
	7	23	S, EC, T	II	13	HD (alcohol)	Chemo	ANED
	8	21	S, IT	IV	120	HD (alcohol)	–	ANED
Morris et al. 2000 [35]^b^	9	33	EC	I	40	NA	–	ANED
	10	24	EC, T	II	55	NA	–	ANED
	11	22	S, CC	I	107	HD (early cirrhosis)	–	NA
Funahashi et al. 2005 [31]^c^ Wymer et al. 2017 [18]^d^	12	30	S, T, IT	I	20	NA	–	ANED
	13	30	T	I	18	NA	–	ANED
	14	35	EC, YST, T	I	11	NA	–	ANED
	15	28	EC, YST, T	II	13	NA	–	ANED
	16	18	EC, YST, T	I	11	NA	Chemo	ANED
	17	32	T, EC, CC	I	15.8	NA	Chemo	ANED
	18	21	EC	I	9.4	NA	PRLND	ANED

*ANED* Alive with no evidence of disease, *CC* Choriocarcinoma, *Chemo* Chemotherapy, *EC* Embryonal carcinoma, *HD* Hepatic dysfunction, *IT* Immature teratoma, *NA* Not available, *PRLND* Retroperitoneal lymphadenectomy, *S* Seminoma, *T* Teratoma, *YST* Yolk sac tumor.

^a, b, c^ One case in each article is seminoma, respectively

^d^ Four cases in the article is seminoma

^e^ All the patients are male, except this patient is female

**Table 2:** Literature review of all previous cases of false elevations of AFP in seminoma

Author	Year of publication	Cases	Stage	Maximum AFP level (ng/ml)	Etiology	Additional Treatment	Follow up
Norgaard-Pedersen et al. [33]	1984	3	NA	30	NA	–	ANED
Germ et al. [34]	1986	2	IV	169	HD	–	ANED
Germa et al. [26]	1993	1	II	36	HD	–	ANED
Nazeer et al. [37]	1998	10	NA	16	NA	Yes (*n* = 5)	ANED
Morris et al. [35]	2000	1	I	20	NA	–	ANED
Funahashi et al. [31]	2005	1	I	40	HD	–	ANED
Vazeille et al. [36]	2016	1	I	19	NA	–	ANED
Wymer et al. [18]	2017	4	I	40.2	NA	–	ANED
Dieckmann et al. [32]	2017	5	I: 3, II: 1, R: 1	20.4	NA	Yes (*n* = 1)	ANED

*ANED* Alive with no evidence of disease, *HD* Hepatic dysfunction, *R* Relapse, *NA* Not available

A total of 45 cases of false-positive AFP level have been reported in testicular GCTs (TGCTs), 17 of them in non-seminomatous TGCTs (Table 1) and 28 cases in seminoma (Table 2). Overall, the reported false elevated AFP levels ranged from 9.4–169 ng/ml, and 84.44% (38/45) of the measurements were lower than 50 ng/ml. The most common cause was liver injury, whereas no etiology was found in some cases, especially in seminoma [31, 33–36].

## Discussion

This case series is relevant to raise the awareness on non-malignant etiologies of elevated serum AFP level in MOGCTs to avoid unnecessary chemotherapy and/or surgery. An unsatisfactory decrease in AFP level during tumor treatment may be caused by residual diseases or acquired chemotherapy resistance. In these situations, patients may be subjected to cytoreductive surgery and salvage chemotherapy. However, other causes associated with AFP elevation need to be fully taken into consideration.

False AFP elevation in GCTs refer to elevated serum AFP levels when there is no clinical evidence of any malignant tumor activity, which is rarely reported in MOGCTs. To the best of our knowledge, only one case has been reported; in 1993, Germa et al. reported a 26-year-old woman with YST who underwent a second-look surgery because of a repeated increasing AFP after chemotherapy. However, the surgery did not find any tumors, and the falsely elevated AFP was associated with drug hepatotoxicity due to anesthetic drugs [26]. The other cases of false-positive AFP elevation have been reported in the male counterpart of MOGCTs, TGCTs. The most common cause is liver injury secondary to alcoholism, drugs and hepatic virus infection, often manifesting as abnormal liver function tests [26]. Hepatocellular regeneration may result in an increased AFP level, which could decline to normality with the improvement of liver function. Another non-pathological cause is the hereditary persistence of AFP, characterized by a related family history with no clinical abnormalities [38]. In some cases, no etiology was reported, especially in seminoma [31, 33–36]. Dieckmann et al. reported approximately 2% of pure seminoma patients had a non-pathologic AFP elevation, and this proportion was not different from that of controlled patients with non-malignant urologic diseases [32]. In addition, AFP can be expressed in other malignancies, of which HCC is the most frequent, and some non-tumor diseases, such as Fanconi anemia and ataxia-telangiectasia [39, 40].

The concanavalin A and *Lens culinaris* agglutinin (LCA) affinity assays are two methods that have been reported to be used in determining the etiology of the AFP [29, 30]. The AFP-L1 elevation (LCA-unreactive) is usually seen in chronic hepatitis and liver cirrhosis, while the AFP-L3 (LCA-reactive) is exclusively produced by tumor cells, and AFP-L3% has been listed as a crucial marker for diagnosis of HCC [30]. Kamoto et al. reported 24 out of 25 (96%) patients with non-seminomatous TGCTs had AFP-L3% > 50% [41]. Although this research included a small number of patients, the results suggested that measurement of AFP-L3% may provide additional information, especially when the total AFP level is persistently elevated during the treatment. In case 4, AFP elevation was accompanied with a high level of serum HBV-DNA, and the liver function in this patient had been abnormal previously. We suspected that the AFP elevation was caused by hepatocellular regeneration instead of tumor relapse. AFP-L3% was found to be < 5%, which also supported our suspicions. However, we should note that all examinations have limitations, and this refractory patient is still in close surveillance.

**Fig. 1:**
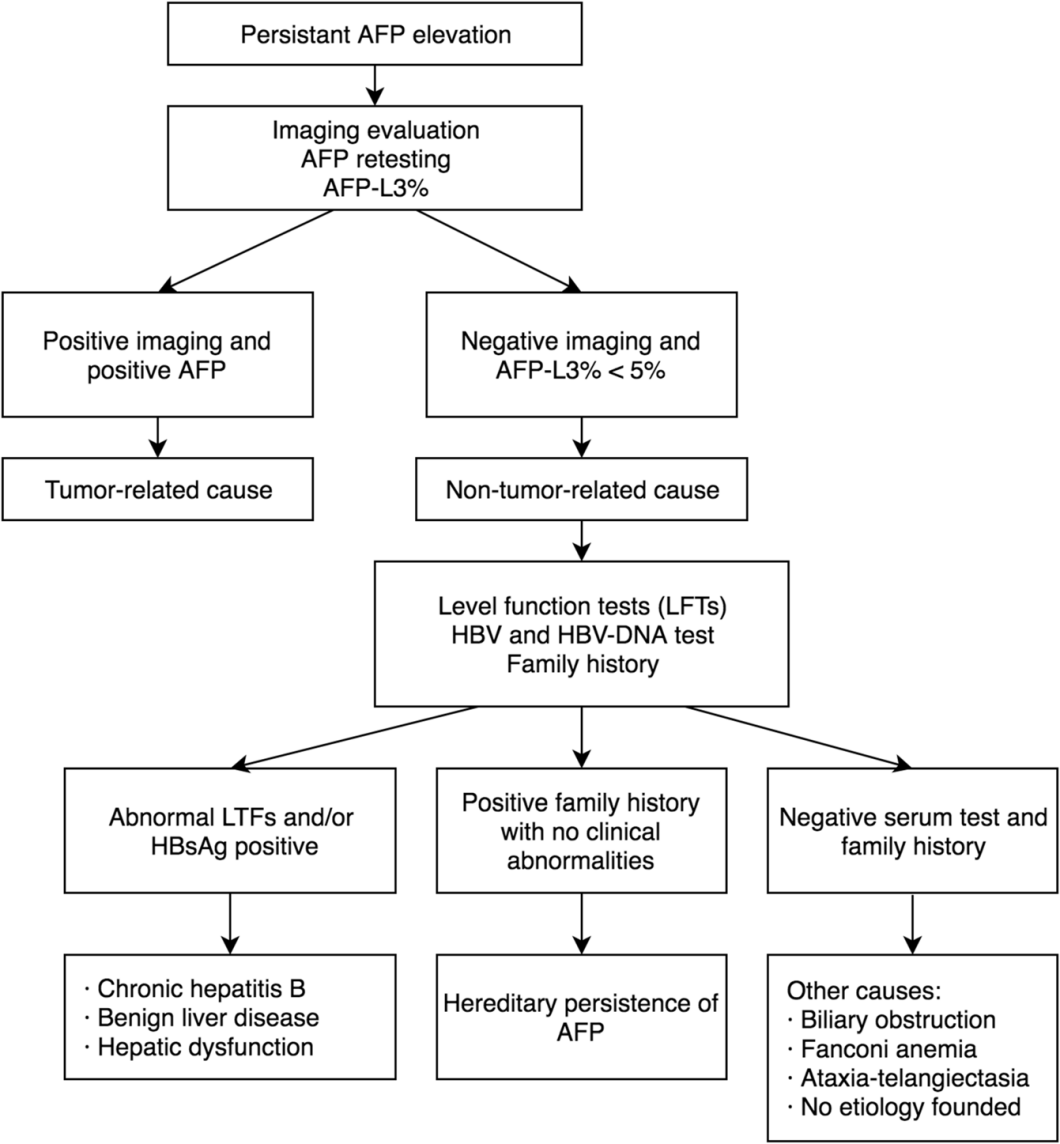
A flow chart of managing persistent AFP elevation in GCTs

AFP is of considerable clinical value during the management of GCTs. AFP, as a typical tumor marker of GCTs, is related to the extent of the disease and can provide useful information for the diagnosis before operation [1]. Serum AFP is highly elevated in YST, usually from thousands up to tens of thousands. AFP elevation can also be seen in other histological subtypes of GCTs, such as IT and embryonal carcinoma, usually from hundreds up to thousands [12, 42]. AFP is supposed to be tested after each cycle of chemotherapy and usually shows a logarithmic decrease trend [13]. When two successive AFP level measurements are similar and persistently elevated, GCT-related causes need to be considered firstly by oncologists. Retesting and imaging evaluation are necessary. AFP-L3% can also be a useful reference index. However, when there is no indication of residual tumors, other conditions associated with AFP elevation need to be considered, especially when AFP is mildly elevated to approximately 100 ng/ml. A flow chart of management of persistent AFP elevation in GCTs is showed in Fig. 1.

Chronic hepatitis B (CHB) is one of the most common non-malignant causes of false positive AFP in GCTs. The mechanism of AFP elevation in CHB has not been fully illustrated. The elevated level of AFP in CHB is usually consistent with the increased serum level of transferase and the presence of bridge fibrosis, which may suggest the association between AFP elevation and hepatic necroinflammatory activity [43]. However, we must emphasize that AFP elevation can also occur in asymptomatic HBsAg carriers with no abnormal liver function tests [44]. As for chronic carriers of HBsAg, replication of HBV may increase during the period of immune suppression. This reactivation of hepatitis B is usually accompanied by the deterioration of liver enzymes, indicating hepatocellular damage and regeneration, which may lead to AFP elevation [45]. As previously reported, AFP level in CHB is usually < 100 ng/ml, and can decline to the normal ranges after the antiviral treatment [44, 46, 47]. In the cases series reported here, all patients were diagnosed with CHB, and the AFP levels were stable and remained at approximately 300 ng/ml during treatment, which is much higher than previously reported levels in literature. The AFP level in case 2 increased during chemotherapy, which may be associated with an increased synthesis of HBV due to bone marrow suppression. The AFP level declined with the recovery of host immunocompetence and antiviral treatments, which further confirms our interpretation.

In clinical practice, the serum AFP level is an important reference index for the termination of chemotherapy. At our medical center, for the initial postoperative chemotherapy strategy, we usually add two more cycles of PEB chemotherapy as consolidation therapy after AFP has decreased to the normal limit. However, as for patients with a persistently elevated AFP level, a comprehensive evaluation is required to identify the cause. In our literature review, at least five cases in non-seminomatous GCTs and six cases in seminoma received additional interventions based on a false elevated AFP level [18, 26, 32, 37]. Albany and Einhorn also suggested that patients with an elevated AFP level should not receive salvage chemotherapy unless there is a sustained rise in AFP [48]. In our second case, the patient could have avoided the last three chemotherapy cycles that were given at a local hospital. A patient with stage I/grade 1 IT does not need to undergo postoperative chemotherapy, but the patient of case 3 was overtreated with PEB chemotherapy due to the abnormal AFP levels. In summary, when there is no indication of residual tumors, patients with elevated AFP should be managed by surveillance instead of additional tumor-related interventions to avoid experiencing unnecessary acute and chronic toxicity.

MOGCTs mostly affect children and women in childbearing age, who have not yet started their families and have a strong desire for fertility preservation [49]. Fertility sparing surgery is feasible, but chemotherapy is inevitably harmful for reproductive ability [50]. What’ more, oncological treatments have a huge impact on patients’ quality of life, mental health and sexual function [51, 52]. Previous studies showed patients with gynecological cancer were at risk of developing psychological problems. A multidisciplinary approach, in which psychologists need to be involved, plays a critical role in providing adequate counselling to support these patients [53].

## Conclusion

The case series presented here demonstrated that a false elevation of AFP could impact management decisions, and a persistently elevated AFP levels in MOGCTs should be assessed by a comprehensive evaluation to avoid unnecessary treatments.

## Data Availability

All data generated and analyzed during this study are included in the published article.

## Abbreviations

AFP: Alpha-fetoprotein
ALT: Alanine transaminase
AST: Aspartate transaminase
CHB: Chronic hepatitis B
DG: Dysgerminoma
GCTs: Germ cell tumors
HBeAg: Hepatitis Be antigen
HBsAg: Hepatitis B surface antigen
HCC: Hepatocellular carcinomas
IT: Immature teratoma
MOGCTs: Malignant ovarian germ cell tumors
PEB: Cisplatin, etoposide, bleomycin
PET/CT: Positron emission tomography/computed tomography
YST: Yolk sac tumor
